# Comparison of the effect of a CIDR-Select Synch versus a long-term CIDR based AI protocol on reproductive performance in multiparous dairy cows in Swiss dairy farms

**DOI:** 10.1186/1477-7827-9-151

**Published:** 2011-11-25

**Authors:** Jürn Rudolph, Rupert M Bruckmaier, Ramanathan Kasimanickam, Adrian Steiner, Marc Kirchhofer, Jürg Hüsler, Gaby Hirsbrunner

**Affiliations:** 1 Food Animal Practice Rudolph, CH-6284 Sulz, Switzerland; 2 Veterinary Physiology, Vetsuisse Faculty, University of Berne, CH-3012 Berne, Switzerland; 3 Department of Veterinary Clinical Sciences, Washington State University, Pullman, WA 99163, USA; 4 Clinic for Ruminants, Vetsuisse Faculty, University of Berne, CH-3012 Berne, Switzerland; 5 Institut of Mathematical Statistics and Actuarial Science, University of Berne, CH-3012 Berne, Switzerland

## Abstract

**Background:**

Synchronization programs have become standard in the dairy industry in many countries. In Switzerland, these programs are not routinely used for groups of cows, but predominantly as a therapy for individual problem cows. The objective of this study was to compare the effect of a CIDR-Select Synch and a 12-d CIDR protocol on the pregnancy rate in healthy, multiparous dairy cows in Swiss dairy farms.

**Methods:**

Cows (N = 508) were randomly assigned to CIDR-Select Synch (N = 262) or 12-d CIDR (N = 246) protocols. Cows in the CIDR-Select Synch group received a CIDR and 2.5 ml of buserelin i.m. on d 0. On d 7, the CIDR insert was removed and 5 ml of dinoprost was administered i.m.. Cows in the 12-d CIDR group received the CIDR on d 0 and it was removed on d 12 (the routine CIDR protocol in Swiss dairies). On d 0 a milk sample for progesterone analysis was taken. Cows were inseminated upon observed estrus. Pregnancy was determined at or more than 35 days after artificial insemination. As a first step, the two groups were compared as to indication for treatment, breed, stud book, stall, pasture, and farmer's business using chi square tests or Fisher's exact test. Furthermore, groups were compared as to age, DIM, number of AI's, number of cows per farm, and yearly milk yield per cow using nonparametric ANOVA. A multiple logistic model was used to relate the success of the protocols to all of the available factors; in particular treatment (CIDR-Select Synch/12-d CIDR), milk progesterone value, age, DIM, previous treatment of the uterus, previous gynecological treatment, and number of preceding inseminations.

**Results:**

The pregnancy rate was higher in cows following the CIDR-Select Synch compared to the 12-d CIDR protocol (50.4% vs. 22.4%; P < 0.0001).

**Conclusion:**

The CIDR-Select Synch protocol may be highly recommended for multiparous dairy cows. The reduced time span of the progesterone insert decreased the number of days open, improved the pregnancy rate compared to the 12-d CIDR protocol and the cows did not to have to be handled more often.

## Background

The average size of a Swiss dairy farm herd is approximately 18 cows [1], compared with herds in the USA, for example, which had an average size of 120 in 2006 [2]. Hence, the use of synchronization programs and fixed-time AI (artificial insemination) is not common. Busato et al., (1995) retrospectively analyzed the health data of 3581 cows from 80 Swiss dairy farms and identified endometritis (19.4%), silent heat (26.4%), non functional ovaries (7.6%) and cystic ovarian disease (5.5%) as the four main fertility disorders [3]. In Swiss dairy farms, the predominant therapy for cows with fertility disorders consists of a CIDR insert for 12 days without administering additional hormones (as described in the Eazy breed™ CIDR^® ^B instruction leaflet), the cows being then inseminated upon observed estrus.

Preparations of Gonadotropine Releasing Hormone (GnRH), human chorionic gonadotropin (hCG), Luteinizing Hormone (LH), and progesterone, either alone or in combination are frequently used to treat anestrus and/or COD [4-8]. Progesterone released from a CIDR insert inhibits the release of GnRH from the hypothalamus and therefore causes GnRH to accumulate in the hypothalamus; removal of the CIDR insert after 12 d causes a large amount of GnRH to be released and this results in ovulation [9-11]. However, the oocyte quality in 12-d CIDR protocols is poor [12]. Different studies showed an acceptable pregnancy rate after the use of a 7-d CIDR protocol in combination with GnRH and/or prostaglandin F_2α _(CIDR-Select Synch) [13-15]. The objective of this study was to compare the effect of a CIDR-Select Synch and a 12-d CIDR protocol on the pregnancy rate in multiparous dairy cows in Swiss dairy farms. Based on a power analysis assuming a 10% increased pregnancy rate (from 35% to 45%) in the CIDR-Select Synch group, we needed a sample size of 380 cows per group to achieve 80% power for a two-sided hypothesis (α = 5%).

## Methods

### Animal selection criteria

Multiparous dairy cows at least 42 DIM (days in milk) of the breeds Holstein Friesian, Red Holstein, Brown Swiss and their crossbreds were included. The veterinarians performed a gynecological examination and cows diagnosed with anestrus, repeat breeders or cows diagnosed as not pregnant after a pregnancy check were included, if the uterus and uterine discharge were normal. Exclusion criteria were a preceding cesarean section, uterine torsion, uterine prolapse or birth-associated injuries of the genital tract. Also, cows with a history of lameness, acute mastitis or any systemic illness within 14 days prior to the CIDR insert were excluded. Any treatment within these 14 days resulted in exclusion. From CIDR removal to the point of insemination no therapy was allowed. Insemination had to be performed within 120 h after CIDR removal. Heifers and cows in first parity were excluded. Cows from farms using bulls were also excluded.

### Treatment and insemination

**Figure 1:**
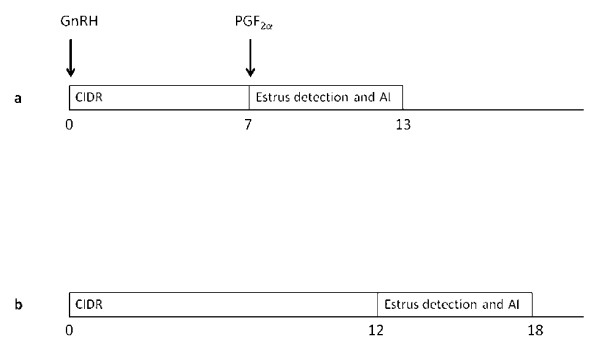
**Schematic representation of the 2 synchronization protocols**. Cows in the CIDR-Select Synch group received a CIDR insert (CIDR; Eazi-Breed CIDR^® ^cattle insert, Pfizer Animal Health, Zurich, Switzerland) and 10 μg of buserelin i.m. (GnRH; Receptal^®^, Veterinaria AG, Zurich, Switzerland) on Day 0. On Day 7, the CIDR insert was removed and 25 mg of dinoprost i.m. (PGF_2α_; Dinolytic^®^; Pfizer Animal Health, Zurich, Switzerland) was administered. The cows were inseminated at observed estrus according to AM-PM rule up to 120 h from CIDR removal (a). Cows in the 12-d CIDR group received a Controlled Internal Drug Release insert (on Day 0) and it was removed on Day 12. The cows were inseminated at observed estrus using AM-PM rule up to 6 d from CIDR removal (b).

Cows were randomly assigned to CIDR-Select Synch (N = 262) or 12-d CIDR (N = 246) protocols based on the cows' odd or even ear-tag numbers, respectively. Cows in CIDR-Select Synch group received a CIDR insert (Eazi-breed™ CIDR^® ^B containing 1.9 g progesterone, Pfizer Animal Health, Zurich, Switzerland) and 2.5 mL of buserelin i.m. (Receptal^® ^4 μg/mL, Veterinaria AG, Zurich, Switzerland) on d 0. On d 7, the CIDR insert was removed and 5 mL of dinoprost i.m. (PGF_2α_; Dinolytic^®^, 5 mg/mL, Pfizer Animal Health, Zurich, Switzerland) was administered. Cows in the 12-d CIDR group received a CIDR insert on d 0 and it was removed on d 12 (Figure 1). They were inseminated at observed estrus according to the AM-PM rule. The cows in both groups were observed for estrus three times daily for a minimum of 30 min. If no estrus was observed within 72 h after CIDR removal, each cow was examined by a veterinarian to exclude silent heat. Silent heat was defined by the presence of a mature follicle (≥ 13 mm in size), a regressing or no CL on one of the ovaries and a strong uterine tone. Cows found to be in silent heat were also inseminated. Cows were observed for estrus and inseminated up to 120 h from CIDR removal.

### Milk progesterone test

A milk sample was collected from each cow on d 0 of treatment. About 5 mL was collected in a plastic tube containing 30 mg of sodium azide (Sigma-Aldrich, Bern, Switzerland) and was frozen until further analysis. The samples were centrifuged for 15 min at 1700 × g and the fat layer was removed. This step was repeated, and skimmed milk samples were further tested. The progesterone concentrations were determined in skimmed milk by enzyme immunoassay as described by Meyer et al. (1986) [16]. The sensitivity of the test was 0.1 ng/mL. Intra- and inter-assay coefficients of variation were 8 and 12%, respectively. Cows with a progesterone level > 1 ng/mL were considered as having luteal activity. A progesterone level < 0.5 ng/mL was considered as representing no luteal activity and the values in between belonged to the third group (indistinct luteal activity).

### Pregnancy determination and definition of pregnancy rate

Pregnancy was determined by rectal palpation and/or transrectal ultrasound at or later than 35 days after artificial insemination. Pregnancy rates per group were determined by calculating the number of cows diagnosed as pregnant following the AI (after the protocol) divided by the total number of cows in the corresponding group. Those cows diagnosed as pregnant that were inseminated more than 6 d after CIDR removal were retrospectively excluded from the analysis.

### Statistical analysis

Data were analyzed with a statistical software program (SAS Version 9.12). The primary end point was 'pregnancy after treatment'. As a first step, the baseline of the two groups were compared as to indication for treatment (non cyclic/negative pregnancy check/signs of estrus), breed (Brown Swiss, Holstein Friesian, Red Holstein and crossbreds), stud book (yes/no), type of stall (freestall/tiestall), pasture (yes/no), and farmer's business (regular/sideline) using chi square tests or Fisher's exact tests. The groups were also compared based on age, DIM, number of AI's, number of cows per farm, and yearly milk yield per cow using nonparametric ANOVA. The second step was to use a multiple logistic model to relate the success of the protocols to all of the available factors; in particular treatment (CIDR-Select Synch/12-d CIDR), milk progesterone value (< 0.5/between 0.5 to 1/> 1 ng/mL), age (< 3.5 yrs/between 3.5 to 5.5 yrs/> 5.5 yrs), DIM (> 100 d; ≤ 100 d) previous treatment of the uterus (yes/no), previous gynecological treatment (yes/no), and insemination (farmer's observation/checked by veterinarian). We used a forward selection procedure. A test result was considered significant if the resulting p-value was < 0.05.

## Results

**Figure 2:**
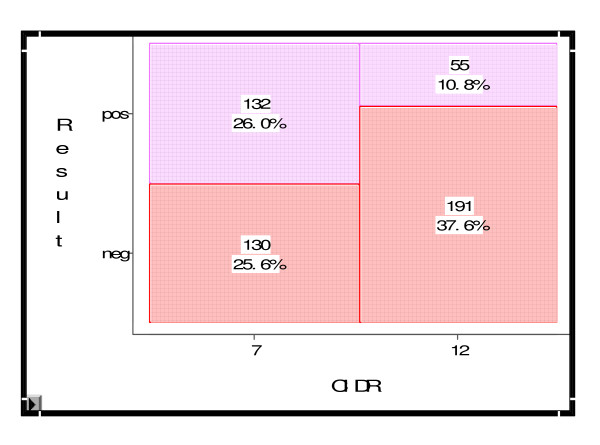
**Mosaic plot of the primary endpoint "confirmed pregnancy"**. Graphic presentation of the percentage of cows in the groups CIDR-Select Synch and 12-d-CIDR with a negative/positive pregnancy test.

Sixteen Swiss practices participated in this study. Multiparous dairy cows (N = 552) were included from April 2009 to August 2010 following routine reproductive checks. The study was brought to an end because of the expiry date of the pharmaceuticals and as the time slot of data acquisition had elapsed. Forty-four cows were retrospectively excluded from the study due to the following reasons: lost CIDR (n = 6), wrong group assignment (n = 10), developing lameness (n = 5), developing vaginitis (n = 6), developing mastitis (n = 5), mounted by bull (n = 5), culling (n = 7). The only significant effect (p < 0.0001) was observed for the CIDR-Select synch treatment (132 cows pregnant of 262) versus CIDR 12-d (55 cows pregnant of 246) with 28% more cows becoming pregnant following the CIDR-Select Synch protocol [with 95% confidence interval (0.199, 0.358)] (Figure 2).

No significant differences (p > 0.1) were found between groups based on: indication for treatment, breed, stud book, type of stall, pasture, farmer's business, age of the cows, DIM, number of AI's before treatment, number of cows per farm, yearly milk yield per cow, milk progesterone value, previous treatment of the uterus, previous gynecological treatment, and characteristic of insemination (Tables 1 and 2). When the cows were grouped according to age, previous gynecological or uterine treatment, DIM, and milk amount, no significant difference was found as to the pregnancy outcome (tested for every variable, without splitting the groups CIDR-Select Synch and 12-d CIDR). There were 78.7% (196/249) of cows in CIDR-Select Synch and 77.3% (157/203) in 12-d CIDR group observed in estrus (P < 0.0001; estrus observed vs. silent heat in both groups). Using the multiple logistic model with the forward selection method, none of the factors exceeded the significant level except for the factor CIDR.

**Table 1:** Baseline comparison of groups at farm level

	CIDR-Select Synch	CIDR12	p-value
*Number of cows*	262	246	**0.224**
*Age of cows (years)*	5 (4/7	5 (4/6.5)	**0.627**
*DIM*	98.5 (71/142)	100 (75/133)	**0.709**
*Number of AI's*	0 (0/1)	0 (0/1)	**0.942**
*Number of cows/farm*	24 (18/32)	25 (19/35)	**0.224**
*Yearly milk yield/cow (kg)*	7500 (6800/8050)	7300 (6800/8300)	**0.554**

There were no significant differences found between the two treatments for the parameters listed (median values (25%/75%), skewed distribution).

**Table 2:** Baseline comparison of groups at cow level

	CIDR-Select Synch	CIDR12	p-value
***Indication for CIDR***			**0.677**
*Acyclic*	71.4%	72%	
*Neg. preg. check*	22.9%	24%	
*No AI*	5.7%	4%	
***Breed***			**0.364**
*Brown Swiss*	40.9%	38.2%	
*Holstein Friesian*	24%	20.7%	
*Red Holstein & crossbreds*	35.1%	41%	
***Studbook***			**0.341**
*Yes*	79.5%	82.8%	
*No*	20.5%	17.2%	
***Type of stall***			**0.542**
*Freestall*	39.5%	36.9%	
*Tiestall*	60.5%	63.1%	
***Pasture***			**0.285**
*Yes*	91.9%	94.3%	
*No*	8.1%	5.7%	
***Farmer's business***			**0.736**
*Regular*	95.7%	95.1%	
*Sideline*	4.3%	4.9%	
***Uterine treatment***			**0.275**
*Yes*	13.4%	10.2%	
*No*	86.6%	89.8%	
***Gynecological treatment***			**0.174**
*Yes*	10.3%	6.9%	
*No*	89.7%	93.1%	
***Insemination***			**0.733**
*Blind*	78.7%	77.3%	
*Checked*	21.3%	22.7%	
***Progesterone value***			**0.223**
*< 0.5 ng/ml*	44.3%	39.4%	
*0.5-1 ng/ml*	14.9%	12.2%	
*> 1 ng/ml*	40.8%	48.4%	

No significant differences were found between the two treatments for the parameters listed (percental distribution).

## Discussion

In Swiss dairy farms, synchronization protocols are more often used to treat individual animals than to synchronize herds. Fertility treatments have to be economical and practical to implement and acceptable to farmers. The CIDR-Select Synch is a viable alternative to the 12-d CIDR protocol because the cows do not need to be handled more frequently. The costs of the additional drugs were expected to be compensated for by the reduction in days open (extending the voluntary waiting period costs 10-20 Swiss francs/cow/day (personal communication Berger & Lauener)). Our study revealed a pregnancy rate for the cows in the CIDR-Select Synch group of 50.4% versus 22.4% in the 12-d CIDR group. Chebel et al., (2010) demonstrated that the use of a CIDR insert for 7 days during a TAI protocol increased the proportion of functional CL in anestrus cows after AI and pregnancy/AI compared to protocols without CIDR [17]. Lamb et al. described a better pregnancy rate in a Cosynch-CIDR protocol compared to Cosynch alone, but only in acyclic cows or cows with a low progesterone level when PGF was administered [18]. The use of exogenous progesterone over a longer period (as described in the Eazy breed™ CIDR^® ^B instruction leaflet) might lead to the ovulatory follicles becoming persistent and to the ovulation of an excessively aged oocyte with a concurrent drop in the pregnancy rate [19]. As a consequence, Swiss practitioners using the 12-d CIDR protocol often skip the CIDR-provoked heat and inseminate cows in the consecutive estrus. The production of remaining large dominant follicles is inhibited by adding GnRH at the beginning of a CIDR protocol, the largest follicles being eliminated by ovulation or atresia [20]. GnRH induces a new follicular wave to emerge within 3-4 days after treatment [20]. Administering GnRH at or after insemination, administering hCG after insemination and supplementing progesterone after insemination have improved reproductive performance in normal, anoestrus and repeat breeder cows [21-25]. In the present study, no hormonal treatment was allowed around insemination. Perry & Perry (2009) demonstrated that treatment with GnRH at AI following the detection of standing estrus in cattle did not influence conception rates [26].

Milk progesterone was used as a covariable in this study and it was measured at d 0 of treatment. The pregnancy rate was not significantly different among the 3 milk progesterone groups (low/intermediate/high) at the beginning of the protocols. Ryan and coworkers (1995) already described this fact in a study using three different CIDR protocols [27]. Progesterone levels were, however, only measured at the beginning of our study.

Ryan et al. (1999) describe an increased estrus detection rate and a decreased CIDR loss rate when the progesterone insertion period was decreased from 12 to 8 days [28]. In both our groups, nearly 80% of cows were inseminated at observed estrus. The estrus detection rate might be better in small herds with moderate milk production as is mostly observed in Switzerland. Lucy described the difficulties of heat detection, identification and insemination in large herds and assumed poor estrus expression as being a major problem in dairy farms with high production levels [29]. Conception rates tended to be higher when AI occurred after detected estrus as compared with fixed-time AI. Pregnancy rates on the other hand, were higher after fixed-time AI when compared with insemination after detected estrus [30].

## Conclusions

The CIDR-Select Synch protocol is a valuable and practical alternative to the 12-d CIDR protocol. Reducing the time span of the progesterone insert also means fewer CIDR inserts lost and fewer days open. We were able to demonstrate that in small herds with intensive observation periods to detect estrus, the CIDR-Select Synch protocol produced a pregnancy rate of 50.4% in multiparous dairy cows. The milk progesterone value at the beginning of the protocol did not significantly influence the outcome.

## Abbreviations

CIDR: Controlled intravaginal drug release; AI: artificial insemination; COD: cystic ovarian disease; GnRH: Gonadotropine Releasing Hormone; hCG: human chorionic gonadotropin; LH: Luteinizing Hormone; PGF: prostaglandin F; DIM: days in milk; TAI: timed artificial insemination.

## Competing interests

The study was financially supported by Pfizer Animal Health, Zurich, Switzerland and Veterinaria AG, Zurich, Switzerland. Before the beginning of the study, publication of data was bound by contract whatever the results would prove.

## Authors' contributions

This study represents the doctoral thesis of JR. JR had the basic idea of performing this study. RB helped with milk progesterone analysis. RK sensitized JR to synchronizing studies and revised the manuscript critically. AS and RK revised the manuscript critically and gave final approval of the version to be published. MK participated in the study design. JH performed the statistical design and analysis including calculating the sample size. GH made substantial contributions to the conception and the design of the study as well as the overall crosslinking. All authors read and approved the final manuscript.

## References

[B1] Agricultural report 2010 http://www.blw.admin.ch/dokumentation/00018/00498/index.html?lang=en

[B2] Changes in size and location US dairy farms http://www.ers.usda.gov/publications/err47/err47b.pdf

[B3] Busato A Minder C Küpfer U Importance and seasonal pattern of fertility disorders in 80 Swiss dairy farms Epidemiol sante anim 1997 31-32 5

[B4] Garverick HA Ovarian follicular cysts in dairy cows J Dairy Sci 1997 80 995 1004 10.3168/jds.S0022-0302(97)76025-9 9178141

[B5] Osawa T Nakao T Kimura M Kaneko K Takagi H Moriyoshi M Kawata K Fertirelin and buserelin compared by LH release, milk progesterone and subsequent reproductive performance in dairy cows treated for follicular cysts Theriogenology 1995 44 835 847 10.1016/0093-691X(95)00269-E 16727779

[B6] Calder MD Salfen BE Bao B Youngquist RS Garverick HA Administration of progesterone to cows with ovarian follicular cysts results in a reduction in mean LH and LH pulse frequency and initiates ovulatory follicular growth J Anim Sci 1999 77 3037 3042 10568475 10.2527/1999.77113037x

[B7] Tebble JE O'Donnell MJ Dobson H Ultrasound diagnosis and treatment outcome of cystic ovaries in cattle Vet Rec 2001 148 411 413 10.1136/vr.148.13.411 11327649

[B8] Chebel RC Santos JE Cerri RL Rutigliano HM Bruno RG Reproduction in dairy cows following progesterone insert presynchronization and resynchronization protocols J Dairy Sci 2006 89 4205 4219 10.3168/jds.S0022-0302(06)72466-3 17033007

[B9] Odde KG A review of synchronization of estrus in postpartum cattle J Anim Sci 1990 68 817 830 2180878 10.2527/1990.683817x

[B10] Roche JF Synchronization of estrous in heifers with implants of progesterone J Reprod Fertil 1974 41 337 344 10.1530/jrf.0.0410337 4452976

[B11] Lane EA Austin EJ Crowe MA Estrous sychronisation in cattle-Current options following the EU regulation restricting use of oestrogenic compounds in food-producing animals: A review Anim Reprod Sci 2008 109 1 16 10.1016/j.anireprosci.2008.08.009 18786787

[B12] Adams GP Jaiswal R Singh J Malhi P Progress in understanding ovarian follicular dynamics in cattle Theriogenology 2008 69 72 80 10.1016/j.theriogenology.2007.09.026 17980420

[B13] Crane MB Bartolome J Melendez P de Vries A Risco C Archbald LF Comparison of synchronization of ovulation with timed insemination and exogenous progesterone as therapeutic strategies for ovarian cysts in lactating dairy cows Theriogenology 2006 65 1563 1574 10.1016/j.theriogenology.2005.09.003 16229884

[B14] Thatcher WW Moreira F Pancarci SM Bartolome JA Santos JE Strategies to optimize reproductive efficiency by regulation of ovarian function Domest Anim Endocrinol 2002 23 1-2 243 254 10.1016/S0739-7240(02)00160-1 12142241

[B15] El-Zarkouny SZ Cartmill JA Hensley BA Stevenson JS Pregnancy in dairy cows after synchronized ovulation regimens with or without presynchronization and progesterone J Dairy Sci 2004 87 1024 1037 10.3168/jds.S0022-0302(04)73248-8 15259238

[B16] Meyer HHD Güven B Karg H Enzymimmuntest (EIA) auf Mikrotiterplatten zur Progesteronbestimmung in Magermilchproben Wien Tierärztl Mschr 1986 73 86 942382445

[B17] Chebel RC Al-Hassan MJ Fricke PM Santos JE Lima JR Martel CA Stevenson JS Garcia R Ax RL Supplementation of progesterone via controlled internal drug release inserts during ovulation synchronization protocols in lactating dairy cows J Dairy Sci 2010 93 922 931 10.3168/jds.2009-2301 20172212

[B18] Lamb GC Stevenson JS Kesler DJ Gaverick HA Brown DR Salfen BE Inclusion of an intravaginal progesterone insert plus GnRH and prostaglandin F2alpha for ovulation control in postpartum suckled beef cows J Anim Sci 2001 79 2253 2259 11583411 10.2527/2001.7992253x

[B19] Mihm M Baguisi A Boland MP Roche JF Association between the duration of dominance of the ovulatory follicle and pregnancy rate in beef heifers J Reprod Fertil 1994 102 123 130 10.1530/jrf.0.1020123 7799304

[B20] Twagiramungu H Guilbault A L Dufour J J Synchronization of follicular waves with a gonadotropin-releasing hormone agonist to increase the precision of estrus in cattle: a review J Anim Sci 1995 73 3141 3151 8617687 10.2527/1995.73103141x

[B21] Howard JM Manzo R Dalton JC Frago F Ahmadzadeh A Conception rates and serum progesterone concentration in dairy cattle administered gonadotrophin releasing hormone 5 days after artificial insemination Anim Reprod Sci 2006 95 224 233 10.1016/j.anireprosci.2005.10.010 16337349

[B22] Stevenson JS Portaluppi MA Tenhouse DE Lloyd A Eborn DR Kacuba S DeJarnette JM Interventions after artificial insemination: conception rates, pregnancy survival, and ovarian responses to gonadotropin-releasing hormone, human chorionic gonadotropin, and progesterone J Dairy Sci 2007 90 331 340 10.3168/jds.S0022-0302(07)72634-6 17183101

[B23] Thuemmel AE Gwazdauskas FC Whittier WD McGilliard ML Effect of progesterone supplementation in repeat-breeder cattle on conception and plasma progesterone Endocrinol Invest 1992 15 393 396 10.1007/BF03348762 1506623

[B24] Son DS Choe CY Cho SR Choi SH Kim HJ Hur TY Jung YG Kang HG Kim IH A CIDR-based timed embryo transfer protocol increases the pregnancy rate of lactating repeat breeder dairy cows J Reprod Dev 2007 53 1313 1318 10.1262/jrd.19066 17827880

[B25] Kim UH Suh GH Hur TY Kang SJ Kang HG Park SB Kim HS Kim IH Comparison of two types of CIDR-based timed artificial insemination protocols for repeat breeder dairy cows J Reprod Dev 2007 53 639 645 10.1262/jrd.18147 17327684

[B26] Perry GA Perry BL GnRH treatment at artificial insemination in beef cattle fails to increase plasma progesterone concentrations or pregnancy rates Theriogenology 2009 71 775 779 10.1016/j.theriogenology.2008.09.050 19004487

[B27] Ryan DP Snijders S Yaakub H O'Farrell KJ An evaluation of estrus synchronization programs in reproductive management of dairy herds J Anim Sci 1995 73 3687 3695 8655445 10.2527/1995.73123687x

[B28] Ryan P D Galvin A J O'Farrell J K Comparison of oestrous synchronization regimens for lactating dairy cows Anim Reprod Sci 1999 56 153 168 10.1016/S0378-4320(99)00028-7 10497912

[B29] Lucy MC Reproductive loss in high-producing dairy cattle: Where will it end? J Dai Sci 2001 84 1277 1293 10.3168/jds.S0022-0302(01)70158-0 11417685

[B30] Stevenson JS Kobayashi Y Thompson KE Reproductive performance of dairy cows in various programmed breeding systems including OvSynch and combinations of Gonadotropin-Releasing Hormone and Prostaglandin F J Dai Sci 1999 82 505 515 10.3168/jds.S0022-0302(99)75261-6 10194668

